# Molecular mechanism of Epimedium in the treatment of vascular dementia based on network pharmacology and molecular docking

**DOI:** 10.3389/fnagi.2022.940166

**Published:** 2022-08-16

**Authors:** Chenchen Xie, Hao Tang, Gang Liu, Changqing Li

**Affiliations:** ^1^Department of Neurology, The Second Affiliated Hospital of Chongqing Medical University, Chongqing, China; ^2^Department of Neurology, Affiliated Hospital & Clinical Medical College of Chengdu University, Chengdu, China

**Keywords:** Epimedium, vascular dementia, dementia, network pharmacology, molecular docking

## Abstract

**Backgroud:** Vascular dementia is the second most common cause of dementia after Alzheimer’s disease, accounting for an estimated 15% of cases. Recently, Epimedium has attracted great attention for its potential neuroprotective benefit. However, the direct role and mechanism of Epimedium on vascular dementia still lack systematic research. To systematically explore the possible pharmacological mechanism of Epimedium for the treatment of vascular dementia, network pharmacology, molecular docking, combined with experiment validation were conducted.

**Methods:** The bioactive compounds and targets of Epimedium were obtained from the TCMSP database. The potential targets of vascular dementia were identified from the DrugBank, OMIM, Genecards, Therapeutic Target Database, and DisGeNET databases. GO and KEGG pathway analyses were performed. Molecular docking was applied to validate the interaction between active components and hub targets. The bilateral common carotid artery occlusion (BCCAO) method was used for construction of a vascular dementia model in mice. The effects of Epimedium on learning and memory ability were examined by behavioral tests. The mechanisms of the cerebral protective effects of Epimedium were evaluated by WB, RT-PCR, and immunofluorescence.

**Results:** A total of 23 Epimedium active ingredients, and 71 intersecting targets of Epimedium against vascular dementia were obtained. The top five hub targets AKT1, TNF, IL1β, IL6, and MMP9 were identified, and molecular docking showed good binding. GO enrichment showed a total of 602 enrichment results, with 458 (80.56%) key targets mainly focused on biological processes (BP). The response to hypoxia, positive regulation of nitric oxide biosynthetic process, aging, inflammatory response, cellular response to lipopolysaccharide, negative regulation of apoptotic process were well ranked. KEGG pathway enrichment analysis identified the TNF signaling pathway as an important pathway, with the MAPK/extracellular signal-regulated kinase (ERK) and NF-κB signaling pathways as the key pathways involved. Consistently, *in vivo* experiments showed that Epimedium treatment improved learning and memory functions in mice with vascular dementia. In addition, Epimedium attenuated the activation of microglia and astrocytes in the hippocampal region after BCCAO. RT-qPCR and Western blot analysis showed that Epimedium not only affected the expression of AKT, TNF, IL1β, IL6, and MMP9, but also suppressed the TNF signaling pathway.

**Conclusion:** Epimedium may exert a protective effect against vascular dementia through the alleviation of oxidative stress, neuroinflammation, BBB dysfunction, apoptosis through TNF signaling pathway. This study explored the mechanism of Epimedium on vascular dementia systematically through network pharmacological and* in vivo* experiment approach, which provides insight into the treatment of vascular dementia.

## Introduction

Vascular dementia (VaD) is recognized as a neurocognitive disorder, which is explained by numerous vascular causes in the case of the general absence of other pathologies. VaD is characterized by severe impairment of cognitive functions such as memory, attention, executive function, and language, with signs and symptoms of focal neurological impairment (Wolters and Ikram, 2019). It is the second most common type of dementia after Alzheimer’s disease (Santos et al., 2018), with a fluctuating course and a stepwise progression (Miceli et al., 2020). As one of the preventable types of dementia, the pathogenesis of VaD involves apoptosis, inflammatory response, oxidative stress, etc. Currently, cholinesterase inhibitors and glutamate receptor antagonists are used to improve the symptoms of memory loss, while they are not fully adequate to improve the symptoms of clinical VaD from multiple aspects and dimensions (Chang et al., 2016). Traditional Chinese medicine (TCM) has been widely used to treat numerous diseases for more than 2,000 years (Cho et al., 2017). Given the complex pathological mechanisms of VaD, the multiple-targets strategy of TCM has been suggested to be beneficial in preventing the development of VaD (Chang et al., 2016).

Epimedium, a plant in the Berberidaceae family, has a long medicinal history (Deng et al., 2018), contains a variety of flavonoids and has a wide range of biological activities. It has been used to treat infertility, chronic musculoskeletal pain, hypertension, coronary sclerosis, angina pectoris, palpitation, chronic bronchitis, and leukopenia (Zhonghuabencao Compilation Commitee, 1999). An increasing number of recent studies have shown the efficacy of Epimedium-containing formulations in brain diseases, including Alzheimer’s dementia, ischaemic brain diseases, vascular dementia, depression, and aging (Zhang, 1999; Du and Yao, 2000; Yi and Gu, 2012; Lin et al., 2013). Modern pharmacological studies have also demonstrated that Quercetin, luteolin, kaempferol, Icariin, the active components of Epimedium, contribute to the alleviation of VaD (Zhou et al., 2011; Deng et al., 2017; Zhou and Li, 2020; Tan et al., 2022). However, systemic research on the direct role and mechanism of Epimedium in VaD is still lacking.

Based on systems biology and bioinformatics, network pharmacology has a wide range of applications in active ingredient discovery, drug target identification, mechanism research, preclinical studies of efficacy, and safety evaluation. Its holistic and systematic characteristics are compatible with the principles of TCM dispensing (Hopkins, 2008). In this study, we investigated the potential mechanisms of Epimedium for the treatment of VaD through network pharmacology, molecular docking approach, and experimental validation to reveal the active ingredients of Epimedium and their respective mechanisms.

## Methods

### Screening for potential active ingredients and targets of Epimedium

The bioactive ingredients of Epimedium were searched through the Traditional Chinese Medicine Systems Pharmacology Database and Analysis Platform (TCMSP^1^), with its Chinese name “Yinyanghuo” as the keyword for searching. The active ingredients of Epimedium and their corresponding targets were obtained through the screening criteria [oral bioavailability (OB) ≥30% and the drug-likeness (DL) ≥0.18; Zhang et al., 2020]. The Uniprot database^2^ was used to search the gene name of the target protein, and the “Organisms” was set to “Homo sapiens”.

### Screening of vascular dementia related targets

Using the search term “vascular dementia” and setting the species as Homo sapiens, the DrugBank database^3^, the OMIM database^4^ and the GeneCards database^5^, Therapeutic Target Database (TTD^6^) and DisGeNET database^7^ were used to obtain vascular dementia related targets. Active targets retrieved from the Genecards database were collected using a relevance score ≥10 as a screening criterion (Hu et al., 2022).

### The construction of protein-protein interaction network

The Cytoscape 3.8.2 software was used to construct and analyze the “ingredient-target” network diagram of Epimedium, in which nodes represent drugs, active ingredients and genes, and edges represent the relationship between them. The targets of Epimedium and the disease targets of vascular dementia were entered into the VENNY 2.1.0 platform^8^ to obtain the intersecting targets and draw a Wayne diagram. The intersection targets were imported into STRING 11.0 platform^9^, and the species was set as “Homo sapiens” and the “medium confidence” was set as 0.400, and the PPI network was constructed. The topological properties of the network were analyzed by network analyzer in Cytoscape 3.8.2 software, and the targets were ranked according to the node degree value (degree; Huang et al., 2022).

### Gene ontology (GO) and kyoto encyclopedia of genes and genomes (KEGG) pathway enrichment analyses

The obtained core targets were imported into the DAVID database^10^ for GO enrichment analysis and KEGG pathway enrichment analysis, with the species set to “Homo sapiens” and the “PvalueCutoff” set to 0.05. The results of GO enrichment and KEGG pathway enrichment were ranked according to the −log10(P) value.

### Molecular docking

The top five targets in the PPI network were selected as the core targets. The crystal structures of the core targets were downloaded from the RCSB PDB^11^ database, and the target proteins were dehydrated and desolvated by Pymol software. Then Autodock software was used to hydrogenate the target proteins and convert the files into pdbqt format for molecular docking. The 2D structures of the compounds in SDF format were downloaded from the PubChem database^12^. The energy minimization was performed using the Minimize mode of Chem3D software and the file was converted to Mol2 format, and then the Mol2 format was converted to pdbqt format using Autodock software. The molecular docking was performed using Autodock software, and the best docking conformation was selected based on the principle of low energy and reasonable conformation. The two-dimensional map of protein-ligand docking was analyzed by LigPlot software.

### Animals

Male C57BL/6J mice (10–12 weeks old, 20–25 g) were provided by the Experimental Animal Center of Chongqing Medical University. All mice were housed in a 12-h light/dark cycle at a temperature of 22 ± 2°C and 65 ± 5% humidity. This experiment was performed in accordance with the guidelines of the National Institute for Animal Research and was approved by the Animal Experimentation Ethics Committee of the Second Affiliated Hospital of Chongqing Medical University.

### Establishment of the vascular dementia model

A mice model of vascular dementia induced by cerebral ischemia/reperfusion was established by the bilateral common carotid artery occlusion (BCCAO) method. Intraperitoneal injections of ketamine (100 mg/kg) and xylazine (10 mg/kg) were administered to sedate the mice, followed by a supine position on the operating table. Following sterilization of the neck skin, the neck muscles and bilateral common carotid arteries (CCA) were bluntly separated. A pair of gold-plated microcoils (0.50-mm pitch; 2.5-mm total length; 0.18-mm internal diameter) manufactured by Motion Dynamics were wrapped around the bilateral CCAs. All mice were continuously observed throughout the procedure and kept on a warm padded surface.

### Epimedium preparation and treatment

Epimedium extract (LY-0014, Hunan Warrant Pharmaceutial Co., LTD) with 98% purity was purchased and diluted to a concentration of 50 mg/ml with distilled water. All animals were divided into the following six groups: sham group, BCCAO group, BCCAO+control group, BCCAO+Epimedium (50 mg/kg), BCCAO+Epimedium (100 mg/kg), BCCAO+Epimedium (200 mg/kg). Mice in the drug group were administered intragastrically with 50 mg/kg, 100 mg/kg, and 200 mg/kg epimedium daily for 8 weeks. Mice in the BCCAO+control group were treated with the same volume of 0.9% saline daily for 8 weeks.

### Morris water maze (MWZ)

The MWZ was used to examine spatial learning and memory function (MWM) at 8th postoperative week. A circular pool with a height of 50 cm, a diameter of 120 cm, and a water depth of 30 cm was used for MWM. The MWM water temperature was maintained at 22 + 0.5°C. Pool was divided into four virtual quadrants and a circular platform with a 5 cm diameter at the center of the target quadrant which was buried 1 cm below the water surface. Spatial acquisition was performed four times a day consecutively for 5 days. During the place navigation phase, mice were randomly placed in the four quadrants of the MWM. Mice were allowed to free-swim until they escaped to the platform in the target quadrant and the computer immediately started the tracking software (Stoelting, USA). Mice were allowed to stay on the platform for 20 s if they reached the platform within 1 min. If the mouse failed to find the platform within 1 min, it was directed to the platform and allowed to stay there for 20 s. In each test, the time to reach the platform was recorded as the escape latency. After 5 days of training, the platform was removed for detection tracking in order to evaluate spatial memory capacity. The mice were placed in the quadrant opposite to the original platform position, and facing the pool wall in the water. The time the mice spent in the desired quadrant and the number of times they crossed the platform position were recorded.

#### Western blot

Brain tissue from the hippocampal region was extracted promptly after anesthesia at each specific time point after BCCAO surgery. Protein content was detected by applying BCA kit (Beyotime, Shanghai, China). SDS-PAGE gels were used to load and separate protein samples, which were then transferred to polyvinylidene difluoride membranes (Millipore, Inc., USA). After blocking in 5% skim milk for 2 h, membranes were incubated overnight with the following primary antibodies: anti-ERK1/2 rabbit antibody (no. 4695, CST, USA, 1:1,000), anti-p-ERK1/2 rabbit antibody (no. 4370, CST, USA, 1:2,000), anti-p38MAPK rabbit antibody (no. 8690, CST, USA, 1:1,000), anti-p-p38MAPK rabbit antibody (no. 4511, CST, USA, 1:1,000), anti-GADPH mouse antibody (no. 97166, CST, USA, 1:5,000), anti-JNK1/2 (no.ab112501, Abcam, USA, 1:1,000), anti-p-JNK1/2 (no.ab4821, Abcam, USA, 1:1,000). The secondary antibody was then incubated with horseradish peroxidase-conjugated secondary antibody for 1 h at 37°C. The membranes were examined using a gel imaging device (Vilber Lourmat fusion FX 7 Spectra, France) and the results were analyzed using software (FUSION-CAPT, France).

#### Real-time quantitative reverse transcription polymerase chain reaction (RT-qPCR)

A Trizol reagent (Takara Biotechnology, Japan) was used to isolate total RNA from hippocampus, and the RNA concentration was determined using a Nano Drop 2,000 spectrophotometer (Thermo Scientific, Bremen, Germany). Total RNA was then reverse transcribed to cDNA by applying the PrimeScript^TM^ RT kit and gDNA Eraser (TaKaRa). RT-qPCR was performed in an iQ5 gradient real-time PCR detection system (Bio-Rad Co., USA). The cycle threshold (Ct value) of each gene was detected, and the relative mRNA content was normalized to housekeeping GADPH gene and calculated by the 2^−∆∆CT^ method. Primer sequences for each gene are listed in Table 1.

**Table 1:** List of primers used for the RT-qPCR.

**Gene**	**Sequence**	
AKT1	Forward	5’-ACCCAGCAGTATGCCAAGTC-3’
	Reverse	5’-GGAAGTCGCTGGTATTGAGC-3’
TNF	Forward	5’-TGCTGCAGGACTTGAGAAGA-3’
	Reverse	5’-GAGGAAGGCCTAAGGTCCAC-3’
IL1β	Forward	5-CCAAAAGATGAAGGGCTGCT-3’
	Reverse	5-ACAGAGGATGGGCTCTTCT-3’
IL6	Forward	5’-AAAGAGGCACTGGCAGAAAA-3’
	Reverse	5’-TTTCACCAGGCAAGTCTCCT-3’
MMP9	Forward	5’-CGTCGTGATCCCCACTTACT-3’
	Reverse	5’-AACACACAGGGTTTGCCTTC-3’
GADPH	Forward	5’-GGTTGTCTCCTGCGACTTCA-3’
	Reverse	5’-TGGTCCAGGGTTTCTTACTCC-3’

#### Immunofluorescence

Mice were anesthetized and transcardially perfused with 0.9% saline and 4% formaldehyde, respectively. The brains were carefully removed, immediately dehydrated with 15% sucrose, and immersed in 30% sucrose the next day. Coronal brain sections of 10 μm thickness were incubated with 1% Triton X-100 for 30 min at room temperature. After blocking with 5% bovine serum albumin for 1 h at 37°C, sections were incubated overnight at 4°C with anti-GFAP mouse antibody (A00213, BOSTER, Inc.1:300), anti-NeuN mouse antibody (MAB377, Millipore, USA, 1:50) and anti-Iba-1 Chicken antibody (Cat234006, SYSY, Inc., Germany, 1:400). Sections were then incubated with Alexa Fluor@647-conjugated goat anti-chicken IgY (ab150171, Abcam, 1:400), Alexa Fluor 594-conjugated goat anti-mouse IgG (bs-0296G-AF594, Bioss, 1:300), or Alexa Fluor 488-conjugated goat anti-mouse IgG (H + L; SA00006-1, Proteintech, 1:300) and incubated with DAPI (Sigma, USA, 1:200) to stain nuclei. All images were taken with an A1 + R laser confocal microscope (Nikon, Tokyo, Japan).

#### Statistical analyses

All data are expressed as the mean ± SEM and were tested for normality and homogeneity of variance with the Kolmogorov–Smirnov test and Levene’s test. Data were assayed using one-way analysis of variance (ANOVA) followed by the Bonferroni *post-hoc* test. LSD method is used for multiple comparisons between groups. The Kruskal-Wallis H test for multiple independent samples were employed if the data was non-normally distributed. Statistical analyses were performed by SPSS 22.0. Statistical significance was set at *P* < 0.05.

## Results

### Bioactive compounds of Epimedium

By searching the TCMSP database, 130 active ingredients of Epimedium were obtained, and 23 active compounds were screened using OB ≥ 30% and DL ≥ 0.18 as criteria (Table 2).

**Table 2:** Bioactive compounds of Epimedium.

**Mol ID**	**Molecule name**	**OB (%)**	**DL**
MOL000622	Magnograndiolide	63.71	0.19
MOL004367	olivil	62.23	0.41
MOL004388	6-hydroxy-11,12-dimethoxy-2,2-dimethyl-1,8-dioxo-2,3,4,8-tetrahydro-1H-isochromeno[3,4-h]isoquinolin-2-ium	60.64	0.66
MOL004382	Yinyanghuo A	56.96	0.77
MOL004396	1,2-bis(4-hydroxy-3-methoxyphenyl)propan-1,3-diol	52.31	0.22
MOL004386	Yinyanghuo E	51.63	0.55
MOL004391	8-(3-methylbut-2-enyl)-2-phenyl-chromone	48.54	0.25
MOL000098	quercetin	46.43	0.28
MOL004384	Yinyanghuo C	45.67	0.50
MOL004373	Anhydroicaritin	45.41	0.44
MOL001645	Linoleyl acetate	42.10	0.20
MOL000422	kaempferol	41.88	0.24
MOL004425	Icariin	41.58	0.61
MOL004394	Anhydroicaritin-3-O-alpha-L-rhamnoside	41.58	0.61
MOL004380	C-Homoerythrinan,1,6-didehydro-3,15,16-trimethoxy-,(3.beta.)-	39.14	0.49
MOL003542	8-Isopentenyl-kaempferol	38.04	0.39
MOL001510	24-epicampesterol	37.58	0.71
MOL001771	poriferast-5-en-3beta-ol	36.91	0.75
MOL000359	sitosterol	36.91	0.75
MOL000006	luteolin	36.16	0.25
MOL003044	Chryseriol	35.85	0.27
MOL001792	DFV	32.76	0.18
MOL004427	Icariside A7	31.91	0.86

### Identification of therapeutic targets of Epimedium

**Figure 1:**
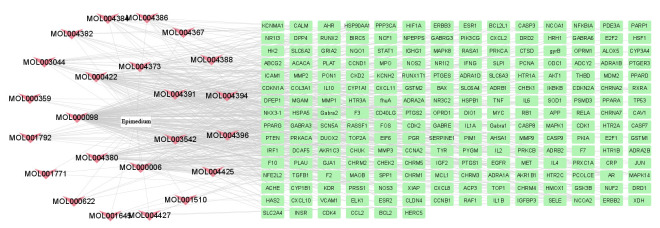
Therapeutic targets of Epimedium. A total of 23 active compounds of Epimedium corresponding to 216 bioactive targets.

A total of 499 gene targets of Epimedium were retrieved from the TCMSP database, and 216 bioactive targets were obtained after removing duplicates. The data were imported into Cytoscape software to construct an ingredient-target network (Figure 1), which contained 240 nodes and 522 edges. The results showed that the top five active ingredients in the degree ranking were MOL000098 (quercetin), MOL000422 (kaempferol), MOL000006 (luteolin), MOL004380 (C-Homoerythrinan, 1,6-didehydro-3,15,16-trimethoxy-, (3.beta.)-), MOL004373 (Anhydroicaritin). They corresponded to 148, 62, 56, 39 and 37 targets, respectively. It indicated that different active ingredients in this network can act on different targets, and the same target can correspond to multiple different active ingredients. It suggested that Epimedium has multi-component and multi-target effects.

### Identification of therapeutic targets of Epimedium in VaD

**Figure 2:**
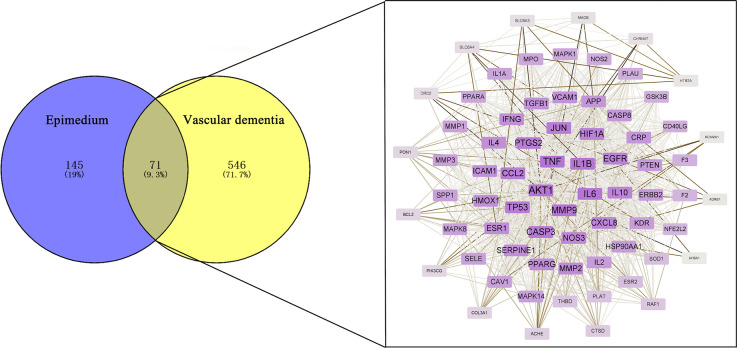
Identification of potential targets for the treatment of VaD by Epimedium. Venn diagram of the overlapping targets of Epimedium and VaD and the PPI network of the 216 targets.

By DrugBank database, OMIM database, GeneCards database, TTD database and DisGeNET database, 1, 118, 396, 0, and 212 targets were obtained respectively, and 617 action targets were obtained after removing duplicates. These targets were matched with 216 targets of Epimedium. Finally, a total of 71 intersecting targets were mapped by the Venny 2.1.0 platform (Figure 2). Among the 23 active ingredients of Epimedium, MOL000098 (quercetin), MOL000006 (luteolin), MOL000422 (kaempferol), MOL004380 (C8-(3-methylbut-2-enyl)-2-phenyl-chromone 8-), and MOL004373 (Anhydroicaritin), corresponding to 57, 24, 21, 14, and 13 intersecting targets between Epimedium and VaD respectively, which suggested an important role of these active ingredients in the treatment of VaD with Epimedium.

The 71 intersecting targets were imported into the String database for protein-protein interaction (PPI) analysis, and the topological analysis of the PPI network was performed by the Network Analyzer of Cytoscape software (Figure 2). The betweenness centrality, closeness centrality, clustering coefficient and degree of topological parameters for each hub gene are shown in Table 2. The top five hub targets in order of degree are Protein Kinase B 1 (AKT1), Tumor Necrosis Factor (TNF), interleukin 1β (IL1β), interleukin 6 (IL6), and matrix metalloproteinase 9 (MMP9; Table 3).

**Table 3:** The topological characteristics of hub targets.

**Hub targets**	**Degree**	**Neighborhood connectivity**	**Betweenness centrality**	**Closeness centrality**	**Clustering coefficient**
AKT1	1.10	0.10	0.91	0.54	63
TNF	1.17	0.02	0.85	0.62	59
IL1B	1.17	0.03	0.85	0.63	58
IL6	1.17	0.03	0.85	0.63	58
MMP9	1.23	0.01	0.81	0.68	55

### Gene ontology enrichment analysis

**Figure 3:**
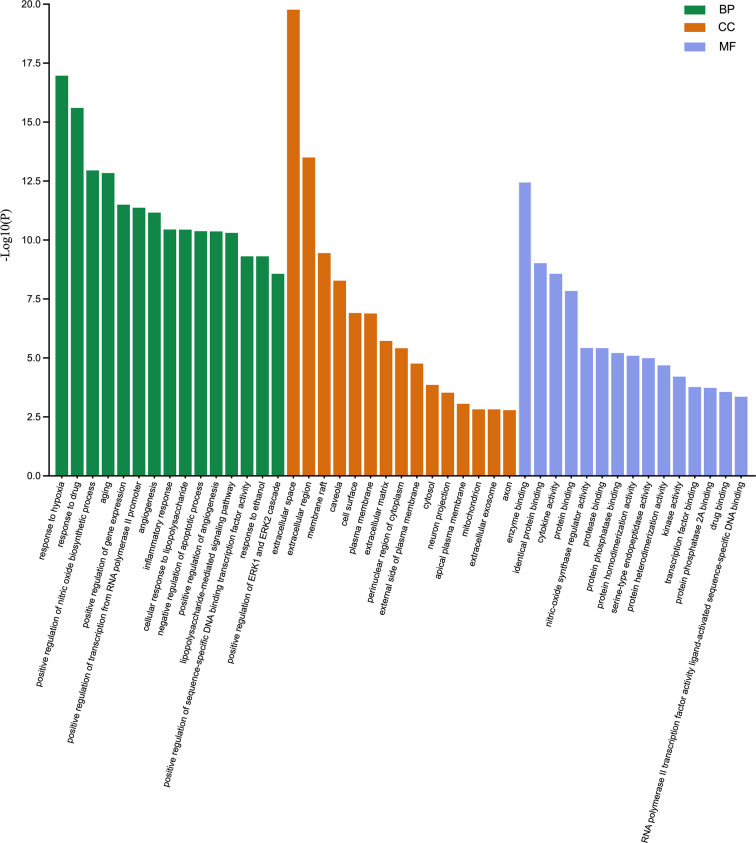
Gene ontology enrichment analysis. The biological process (BP), cellular component (CC), and molecular function (MF) terms identified by GO enrichment analysis are presented by a two-dimensional bar chart according to the −log10(P) value.

**Figure 4:**
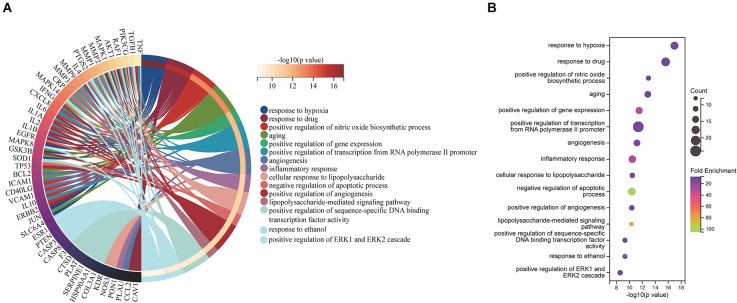
BP associated with hub targets of Epimedium against VaD. **(A)** BP is presented by Circro diagrams. **(B)** BP is presented by bubble diagrams with BP term, −log10(p value), gene count, and fold enrichment.

The 71 intersecting targets were imported into the DAVID database for GO enrichment analysis, The top 15 entries of the ranked GO enrichment rank were selected according to the −log10(P) value to draw a two-dimensional bar chart (Figure 3). The results showed a total of 602 enrichment results, with 458 (80.56%) key targets mainly focused on biological processes (BP). The top 15 biological process (BP) terms identified by GO enrichment analysis (Figure 4) were response to hypoxia, response to drugs, positive regulation of nitric oxide biosynthetic process, aging, positive regulation of gene expression, positive regulation of transcription from RNA polymerase II promoter, angiogenesis, inflammatory response, cellular response to lipopolysaccharide, negative regulation of apoptotic process, positive regulation of angiogenesis, lipopolysaccharide-mediated signaling pathway, positive regulation of sequence-specific DNA binding transcription factor activity, response to ethanol, positive regulation of ERK1 and ERK2 cascade.

**Figure 5:**
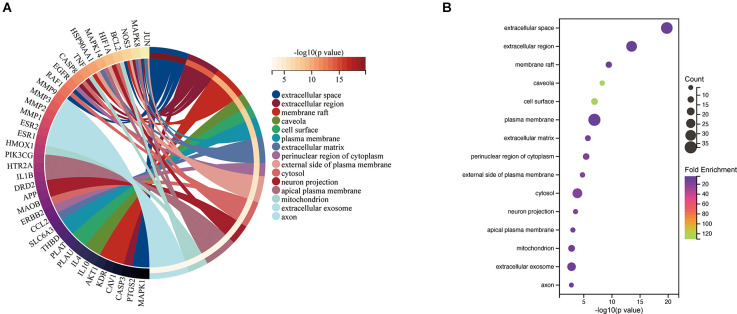
CC associated with hub targets of Epimedium against VaD. **(A)** CC is presented by Circro diagrams. **(B)** CC is presented by bubble diagrams with CC term, −log10(p value), gene count, and fold enrichment.

The top 15 cellular component (CC) terms identified by GO enrichment analysis (Figure 5) were ranked as follows: extracellular space, extracellular region, membrane raft, caveola, cell surface, plasma membrane, extracellular matrix, perinuclear region of cytoplasm, external side of plasma membrane, cytosol, neuron projection, apical plasma membrane, mitochondrion, extracellular exosome, axon.

**Figure 6:**
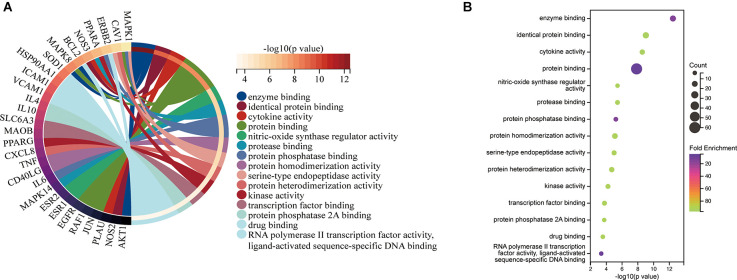
MF associated with hub targets of Epimedium against VaD. **(A)** MF is presented by Circro diagrams. **(B)** MF is presented by bubble diagrams with MF term, −log10(p value), gene count, and fold enrichment.

The top 15 molecular function (MF) terms identified by GO enrichment analysis (Figure 6) were ranked as follows: enzyme binding, identical protein binding, cytokine activity, protein binding, nitric oxide synthase regulatory activity, protease binding, protein phosphatase binding, protein homodimerization activity, serine-type endopeptidase activity, protein heterodimerization activity, kinase activity, transcription factor binding, protein phosphatase 2A binding, drug binding, RNA polymerase II transcription factor activity, and ligand-activated sequence-specific DNA binding.

### KEGG pathway enrichment analysis

**Figure 7:**
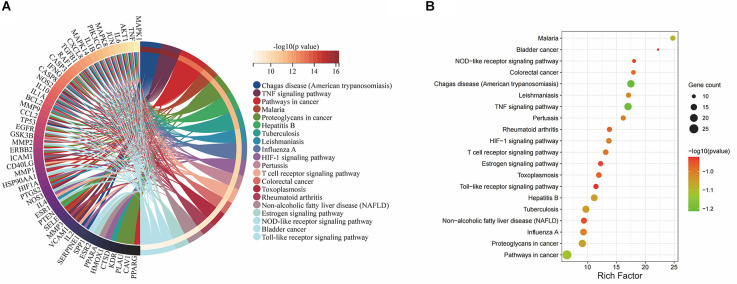
KEGG pathway enrichment analysis. **(A)** The top 20 items ranked by −log10(p) value is shown by the Circro diagrams. **(B)** The top 20 items ranked by −log10(p) value, gene count, and rich factor are shown by the bubble map.

**Figure 8:**
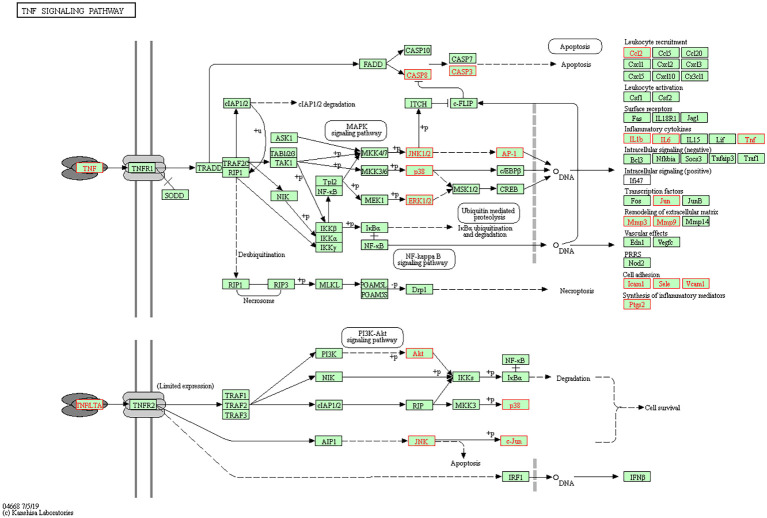
TNF signaling pathway. The red targets are the hub genes which involved in the therapeutic effect of Epimedium on VaD.

The 71 intersecting targets between Epimedium and VaD were imported into the DAVID database for KEGG pathway enrichment analysis, and 150 items were obtained, and the top 20 items ranked by -log10(p) value were taken to plot Circro diagrams (Figure 7A) and the bubble map (Figure 7B). Among them, the TNF signaling pathway was ranked second, and the specific positions of 18 targets (red) involved in the therapeutic effect of Epimedium on VaD were plotted in the TNF signaling pathway map by KEGG mapper platform (Figure 8).

### Results of molecular docking experiments

**Figure 9:**
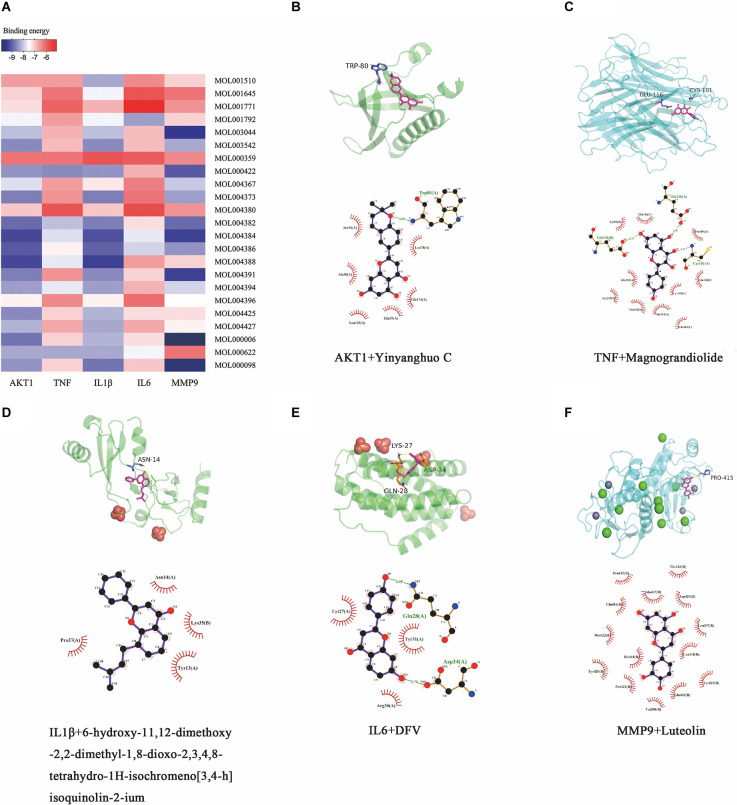
Molecular docking results. Heatmap and molecular docking results of molecular docking. **(A)** The binding energy value of molecular docking. **(B)** AKT1 docked with Yingyanghuo C. **(C)** TNF docked with Magnograndiolide. **(D)** IL1β docked with 6-hydroxy-11,12-dimethoxy-2,2-dimethyl-1,8-dioxo-2,3,4,8-tetrahydro-1H-isochromeno[3,4-h]isoquinolin-2-ium. **(E)** IL6 docked with DFV. **(F)** MMP9 docked with Luteolin.

The top five “degree” targets of AKT1, TNF, IL1β, IL6, and MMP9 were selected for molecular docking with 23 active ingredients of Epimedium (Figure 9A, Table 4). A binding energy less than −5 kJ·mol^−1^ suggested that the target specifically bound the compound (Gaillard, 2018). In this study, Yinyanghuo C, Magnograndiolide, 6-hydroxy-11,12-dimethoxy-2,2-dimethyl-1,8-dioxo-2,3,4, 8-tetrahydro-1H-isochromeno[3,4-h]isoquinolin-2-ium, DVF, and luteolin are used to demonstrate the molecular docking diagram (Figures 9B–F). The output of Pymol software showed that the small molecule active ingredients could bind to amino acid residues such as TRP80, GLU116, CYS101, ASP34, Gln28, LYS27, etc., resulting in hydrogen bonding effect and thus increasing the structural stability. Yinyanghuo C binds to the TRP80 site of AKT1 (Figure 9B). Magnograndiolide binds to the GLU116 and CYS101 sites of TNF (Figure 9C).

**Table 4:** The results of molecular docking (kJ/mol).

**Compound**	**AKT1**	**TNF**	**IL1β**	**IL6**	**MMP9**
24-epicampesterol	−6.6	−6.6	−8.3	−6.4	−7.1
Linoleyl acetate	−7.1	−6.2	−7.6	−5.8	−6.2
poriferast-5-en-3beta-ol	−7.2	−6.0	−6.8	−5.1	−6.5
DFV	−7.6	−6.6	−7.6	−8.4	−7.1
Chryseriol	−8.1	−6.8	−8.1	−6.9	−9.5
8-Isopentenyl-kaempferol	−8.6	−6.9	−8.6	−6.9	−8.3
sitosterol	−6.2	−6.2	−5.8	−5.9	−6.4
kaempferol	−8.5	−8.6	−8.5	−6.8	−9.1
olivil	−7.8	−6.6	−7.3	−6.3	−8.0
Anhydroicaritin	−8.7	−6.5	−8.7	−6.2	−8.4
C-Homoerythrinan,1,6-didehydro-3,15,16-trimethoxy-,(3.beta.)-	−7.0	−5.8	−7.0	−5.7	−6.5
Yinyanghuo A	−8.9	−8.1	−8.7	−7.2	−8.9
Yinyanghuo C	−9.3	−7.8	−9.2	−7.7	−9.4
Yinyanghuo E	−9.1	−7.4	−9	−7.9	−8.5
6-hydroxy-11,12-dimethoxy-2,2-dimethyl-1,8-dioxo-2,3,4,8-tetrahydro-1H-isochromeno[3,4-h]isoquinolin-2-ium	−9.3	−7.7	−9.4	−6.6	−7.1
8-(3-methylbut-2-enyl)-2-phenyl-chromone	−8.7	−6.5	−8.6	−7.1	−9.4
Anhydroicaritin-3-O-alpha-L-rhamnoside	−8.4	−7.7	−8.4	−6.8	−7.9
1, 2-bis(4-hydroxy-3-methoxyphenyl)propan-1, 3-diol	−7.4	−6.4	−7.4	−6.1	−7.5
Icariin	−8.6	−7.1	−8.2	−7.2	−7.2
Icariside A7	−8.2	−6.7	−8.3	−6.7	−7.4
luteolin	−8.8	−7.1	−8.2	−7.2	−9.9
Magnograndiolide	−8.3	−8.3	−8.3	−7.6	−6.2
quercetin	−8.5	−7.1	−8.8	−7.0	−9.7

6-hydroxy-11,12-dimethoxy-2,2-dimethyl-1,8-dioxo-2,3,4,8-tetrahydro-1H-isochromeno[3,4-h]isoquinolin-2-ium binds to the ASN14 site of IL1β (Figure 9D). DVF binds to the ASP34, Gln28, and LYS27 sites of IL6 (Figure 9E), and luteolin binds to the PRO415 site of MMP9 (Figure 9F). It indicates a strong affinity between the active ingredient and the target.

### Epimedium treatment improved learning and memory functions in VaD mice

**Figure 10:**
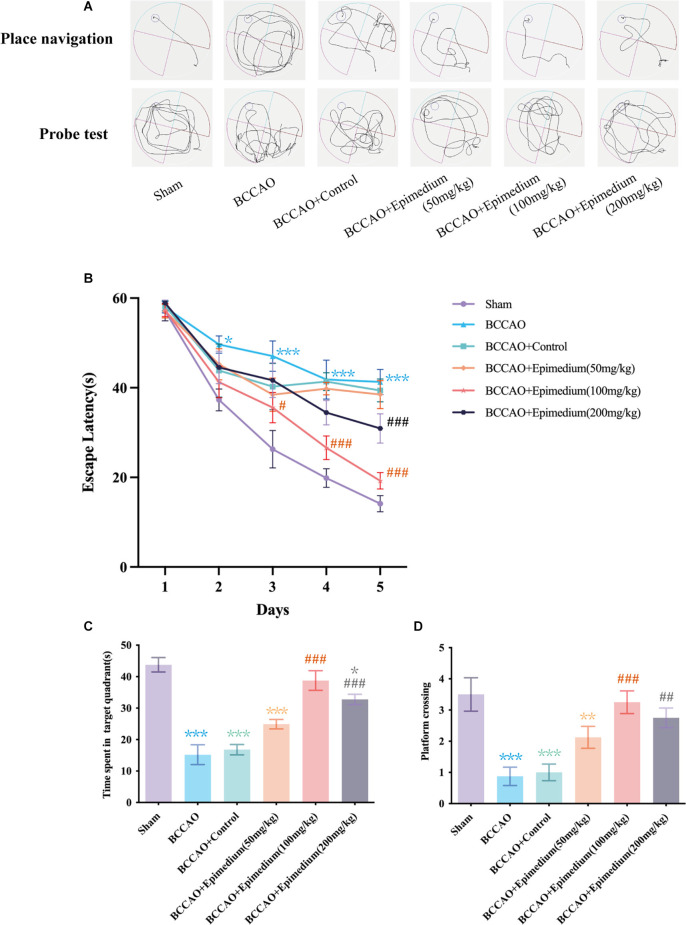
Epimedium treatment alleviated VaD induced cognitive impairment. **(A)** The present track route of place navigation and probe test. **(B)** The escape latency in each group (*n* = 8). **(C)** Time spent in the target quadrant in the probe trial. **(D)** The number of platform crossing in the probe trial. Values are expressed as mean ± SEM (*n* = 8). ***P < 0.001, **P < 0.01 and *P < 0.05 vs. the sham group; ^###^*P* < 0.001, ^##^*P* < 0.01, and ^#^*P* < 0.05 vs. the BCCAO group.

The MWM test is the most widely used behavioral test to evaluate rodent cognitive deficits (Figure 10). In the platform navigation test (Figures 10A,B), the escape latency was significantly prolonged in the BCCAO group compared to the sham group. On days 3 and 5, the escape latency in BCCAO+Epimedium (50 mg/kg), BCCAO+Epimedium (100 mg/kg) and BCCAO+Epimedium (200 mg/kg) was significantly shorter than that in the BCCAO group. Both the time spent in the target quadrant and the number of platform crossing were significantly reduced in the BCCAO group compared with the sham group in the probe test (Figures 10C,D). However, Epimedium treatment at 100 mg/kg and 200 mg/kg improved cognitive impairment. These results suggest that Epimedium treatment can ameliorate VaD-induced learning and memory impairment, and Epimedium at a dose of 100 mg/kg showed to be more effective in the improvement of cognitive impairment.

### Epimedium alleviated microglial and astrocyte activation in VaD mice

**Figure 11:**
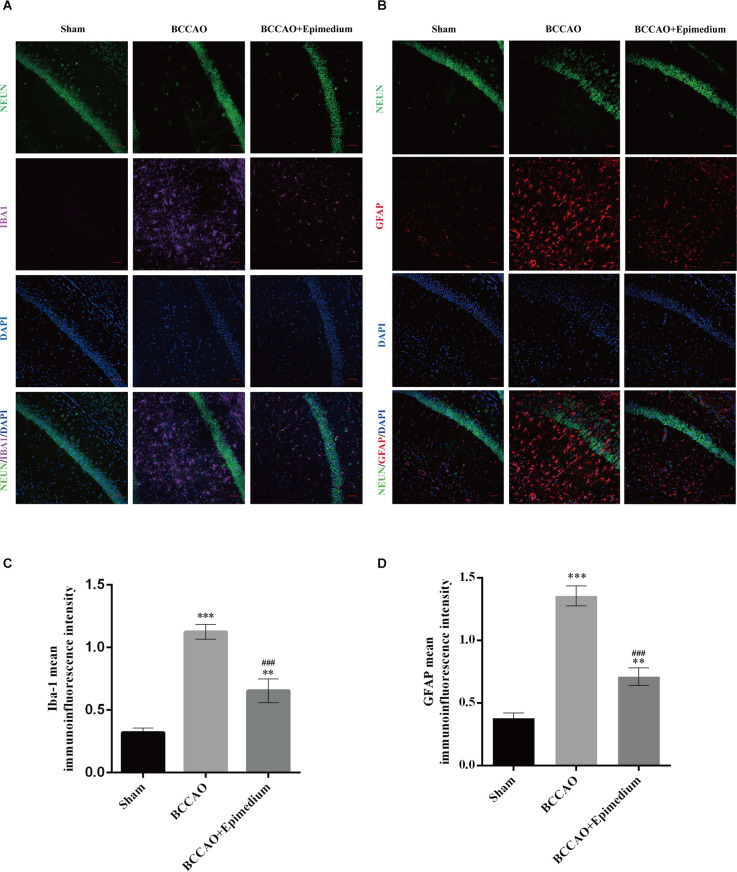
Epimedium alleviated the activation of microglia and astrocytes in the hippocampus in VaD mice. Immunofluorescence was used to stain microglia and neuron **(A)** and astrocytes and neuron **(B)**. Panels **(C)** and **(D)** represent the quantitative analysis of the mean immunoinfluorescence intensity of Iba-1 and GFPA in each group (Scale bar = 50 μm, *n* = 5). ***P < 0.001 and **P < 0.01 vs. the sham group; ^###^*P* < 0.001 vs. the BCCAO group.

The activation of microglia and astrocytes in the hippocampus was measured by immunofluorescence. Microglia in the Sham group were mainly in a resting state with the smaller cell bodies and branches (Figure 11A). In contrast, microglia in the BCCAO group and BCCAO+Epimedium group obtained a reactive phenotype. The relative fluorescence intensity in the BCCAO and BCCAO+Epimedium groups was significantly higher than that in the sham group. Compared with the BCCAO group, the relative fluorescence intensity was significantly decreased by Epimedium treatment (Figure 11C). The relative fluorescence intensity of GFAP in the BCCAO group was significantly higher than that in the sham group. A significant decrease in the relative fluorescence intensity of GFAP was observed in the BCCAO+Epimedium group compared to the BCCAO group after Epimedium treatment (Figures 11B,D).

### Epimedium modulated the inflammation and activation of the TNF signaling pathways in VaD mice

**Figure 12:**
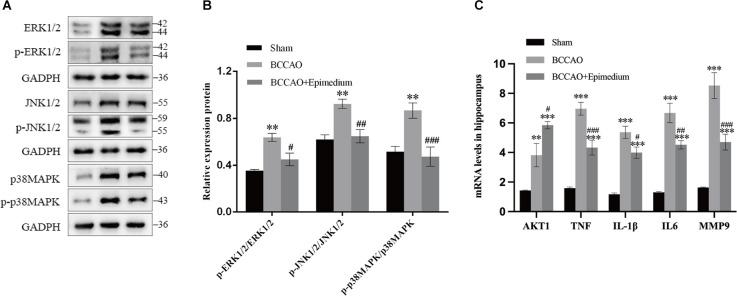
Epimedium modulated the secretion of cytokines and activation of the MAPK/ERK/JNK pathways in VaD mice. The contents of AKT1, TNF, IL-1β, IL-6, and MMP in the hippocampus (**C**, *n* = 5) were analyzed by RT–qPCR. Western blotting was used to detect p-ERK1/2, ERK1/2, p-JNK1/2, JNK1/2, p-p38MAPK, and p38MAPK proteins in hippocampus (**A,B**, *n* = 3). ***P < 0.001 and **P < 0.01 vs. the sham group; ^###^*P* < 0.001, ^##^*P* < 0.01, and ^#^*P* < 0.05 vs. the BCCAO group.

The animals were divided into three groups. Sham group, BCCAO group, and BCCAO+Epimedium group. The inflammation and cell survival-related cytokines AKT1, TNF, IL1β, IL6, and MMP9 were detected by RT-qPCR. In the hippocampus, the mRNA levels of TNF, IL1β, IL6, and MMP9 were significantly increased in the BCCAO group compared with Sham group, however, they were significantly decreased in the BCCAO+Epimedium group. In addition, there were a significant increase in the mRNA levels of AKT in the BCCAO group compared with the Sham group and a further increase in the BCCAO+Epimedium group (Figure 12C).

The TNF signaling-related MAPK pathway in the hippocampus was next analyzed in order to explore the possible mechanisms of Epimedium-mediated neuroprotection following VaD. The protein expression of p-ERK1/2, ERK1/2, p-JNK1/2, JNK1/2, p-p38MAPK, and p38MAPK was detected by Western blotting. P-ERK1/2/ERK1/2, p-JNK1/2/JNK1/2, and p-p38MAPK/p38MAPK ratios were used to indicate the phosphorylation of ERK1/2, JNK1/2, and p38MAPK. As shown in Figures 12A,B, increased phosphorylation ratios of ERK1/2, JNK1/2, and p38MAPK were detected in the BCCAO group compared to the Sham group. The ratio of phosphorylated ERK1/2, JNK1/2, and p38MAPK detected in the BCCAO+Epimedium group was decreased compared to the BCCAO group.

## Discussion

In recent years, the role of Epimedium in the central nervous system has increasingly gained attention and studies have shown that it has a cerebral protective effect (Zhou and Li, 2020). Few studies have shown the efficacy of Epimedium-containing formulations in VaD (Cho et al., 2017). Epimedium combined with Panax notoginseng saponins could significantly improve the spatial learning and memory abilities of VaD model rats (Zheng et al., 2008). Meanwhile, study have showed that the combination of Epimedium and total Saponins of Panax Ginseng can improve oxygen free radical metabolism and neuronal injury in hippocampal CA1 region, which exert a protecting role in VaD (Zheng et al., 2014). However, the direct role and the underline mechanism of Epimedium on VaD still remain unclear. TCM is prominently characterized using multiple components targeting multiple factors to produce a therapeutic effect (Tao et al., 2020). Therefore, direct administration of Epimedium may protect against VaD through multiple targets and pathways, and the exploration of its mechanisms would be valuable. Therefore, this study employed network pharmacology, molecular docking and experimental validation to reveal the underlying role and mechanism of Epimedium in VaD.

In this study, we constructed a vascular dementia BCCAO model and applied different doses of Epimedium as treatment. Based on the results of improved cognitive function, we found that 100 mg/kg of Epimedium had a significant neuroprotective effect. Twenty-three active components of Epimedium were obtained by searching the TCMSP database. After the common targets of Epimedium and VaD were filtered, 71 targets were then screened out and used to construct a PPI network, and the top five hub targets (AKT1, TNF, IL1β, IL6, and MMP9) were ultimately identified. In particularly, quercetin, luteolin, and kaempferol possess relatively more therapeutic targets for VaD. Importantly, molecular docking experiments further revealed that most of the bioactive components of Epimedium exhibit strong affinity for the top five hub targets, suggesting the therapeutic effects of Epimedium on VaD.

Cerebral hypoperfusion, oxidative stress and neuroinflammation play pivotal role in the development of VaD, which lead to endothelial damage, disruption of trophic coupling between vascular and brain cells, blood-brain barrier (BBB) breakdown, neuronal apoptosis (Pimplikar, 2009). In this study, the biological process (BP) terms identified by GO enrichment analysis of the hub genes showed response to hypoxia, positive regulation of nitric oxide biosynthetic process, aging, inflammatory response, negative regulation of apoptotic process, positive regulation of ERK1 and ERK2 cascade. These results are consistent with the pathological processes of VaD. Oxidative stress occurs when the balance of reactive oxygen species (ROS) and antioxidants disrupted and is increasingly implicated in VaD (Choi et al., 2014). Oxidative stress can cause DNA damage and induce oxidation of proteins and that lipids eventually results in apoptosis (Yamagishi et al., 2008). Quercetin, as a component of Epimedium, has been shown to reduce oxidative stress, decrease NO levels, and inhibit NOS activity in brain tissue. It exerted a protective role on cognitive function in VaD by improving cerebral blood flow, regulating oxidative stress, cerebral energy metabolism and cholinergic function (Tota et al., 2010; Khan et al., 2021; Singh and Garabadu, 2021). Researches also have found that luteolin, another component of Epimedium, can inhibit cerebral ROS levels, and modulate inflammatory factors to achieve protective effects on neurons through anti-oxidative stress, anti-inflammatory and anti-apoptotic effects (Bastianetto et al., 2011; Wang and Gao, 2015). Icariin can improve spatial learning and memory impairment by scavenging free radicals, improving anti-lipid peroxidation, inhibiting neuronal apoptosis (Zheng et al., 2008). Mounting evidence showed that Aβ has powerful vascular effects during the development of VaD (Richard et al., 2010). It was found that kaempferol is a potent inhibitor of Aβ aggregation which ameliorates Aβ-induced neuronal cell death, ROS production (Wang et al., 2016; Yang et al., 2016; Airoldi et al., 2018).

KEGG pathway analysis of the present study revealed that the TNF signaling pathway was ranked the second, and the specific positions of 18 targets involved in VaD were plotted in the TNF signaling pathway, involving the top five targets AKT1, TNF, IL1β, IL6, and MMP9. TNF signaling pathway, a key pathway that initiates the inflammatory response, has significant pro-inflammatory and pro-apoptotic effects that induce the development of VaD. TNF signaling pathway can further regulate MAPK, NF-κB, and PI3K/AKT signaling pathway to mediate inflammatory response, apoptosis, and cell survival (Xu et al., 2021). PI3K/Akt signaling pathway is an important signaling pathway that regulates cell survival and activated Akt plays a key role in anti-apoptosis (Xu et al., 2021). We found that Epimedium can improve the expression of AKT, which may involved in the anti-apoptosis effect in VaD.

Neuroinflammation is an important factor in the progression of VaD (Rosenberg, 2009), as increased levels of systemic pro-inflammatory cytokines in VaD patients have been widely reported (Schmitz et al., 2015; Belkhelfa et al., 2018). Neuroinflammation is typically characterized by increased production of pro-inflammatory cytokines and chemokines by resident brain cells such as microglia and astrocytes, along with infiltration of peripheral immune cells into the central nervous system, which is usually in response to bioenergetic imbalance and oxidative stress (Newton and Dixit, 2012). Our present study also showed significant activation of microglia and astrocytes in the hippocampus. The molecular function (MF) terms showed enzyme binding, identical protein binding, cytokine activity, protein binding, nitric oxide synthase regulatory activity, which are involved throughout the neuroinflammatory response. Studies reported elevated levels of inflammatory factors such as TNFα, IL-1β, and IL-6, leading to degradation of the tissue matrix and infiltration of peripheral immune cells causing cell death (Engelhart et al., 2004). The development of VaD can also be promoted by these pro-inflammatory cytokines through activation of microglia, the neuronal damage *via* oxidative stress, and direct damage to cerebral white matter and induction of cerebral microvascular endothelial dysfunction (Wang et al., 2017). Consistent with these studies, we found significant increses in IL-1β, TNF, IL-6, and IL-1β in the hippocampus following VaD. Epimedium treatment obviously suppressed the expression of IL-1β, TNF, and IL-6 and the activation of microglia and astrocytes, which indicates that the neuroprotective effect of Epimedium is mediated by regulating neuroinflammation in VaD. Quercetin, an important component of Epimedium, can improve cognitive impairment and enhance memory during VaD (Jakaria et al., 2019). It facilitated secretion of anti-inflammatory cytokines (IL-4 and IL-10) and in turn decreased production of pro-inflammatory factors (TNF-α and IL-1β) due to regulating microglial phenotype transformation (Tan et al., 2022). Its strong antioxidant and anti-inflammatory properties can effectively inhibit oxidative stress-mediated neuronal damage and down-regulate the expression of chemokines and pro-inflammatory cytokines to reduce the inflammatory response of neurons (Suganthy et al., 2016; Yang et al., 2020). Icariside, another component of Epimedium, can also improve cognitive dysfunction by inhibiting inflammatory factor expression, suppressing activation of microglia and astrocytes, and preventing neuronal apoptosis (Deng et al., 2017). It was found that kaempferol can alleviate the release of inflammatory factors, microglia activation, and modulation of MAPK, NF-κB signaling pathway (Wang et al., 2016; Yang et al., 2016; Airoldi et al., 2018). The pro-inflammatory cytokine TNF-α is an important ligand for the death receptor and activates pro-apoptotic caspase-8 and -3 in the apoptotic pathway during VaD (Micheau and Tschopp, 2003). According to the KEGG results, Epimedium may inhibit apoptosis mediated by caspase-8 and caspase-3 by regulating the TNF signaling pathway. Zhou et al. found that Luteolin may protect neurons from ROS-mediated apoptosis by inhibiting the NF-κB, MAPK pathway, which would ameliorate the progression of VaD (Zhou et al., 2011). Importantly, this study showed that Epimedium inhibited the activation of TNF and MAPK/ERK pathways. Accordingly, it makes sense to believe that Epimedium reduces the production and release of pro-inflammatory factors by inhibiting TNF related signaling pathways. In the follow-up study, we will apply antagonists or genetic interventions to interfere with these pathways to clarify the direct role of TNF-related pathways that are involved in the neuroprotective effect of Epimedium in VaD.

The acute and chronic neuroinflammation of VaD can damage the BBB, myelin sheath, and gray matter *via* oligodendrocyte loss, endothelial cell dysfunction, destruction of the neurovascular unit (Zhou et al., 2017; Miyanohara et al., 2018; Poh et al., 2021). BBB dysfunction is increasingly implicated in VaD (Wardlaw et al., 2003). VaD-induced oxidative stress and inflammation promote BBB dysfunction significantly by decreasing the density of tight junction proteins (TJPs; Hill et al., 2014). The proinflammatory cytokines can increase the expression of matrix metalloproteinases (MMPs), which can also degrade the extracellular matrix contributing to BBB dysfunction (Blamire et al., 2000; Ihara et al., 2001). As our study showed, Epimedium may regulate the inflammatory response and modulate the expression of MMPs through the TNF signaling pathway, thus aggravating the BBB dysfunction. In the animal experiment, we futher clarified that MMP expression was increased in VaD, which may be involved in the disruption of the BBB. In contrast, Epimedium decreases the expression of MMP9, which may have a protective effect on the BBB. In addition, axonal damage may be a major cause of cognitive dysfunction in VaD (Raff et al., 2002). Inflammation activates glial cells to release pro-inflammatory factors such as TNF-α, MMPs that damage the myelin sheath (Chandler et al., 1995). Moreover, the inflammation during VaD can initiate the apoptosis and pyroptosis of oligodendrocytes (Márquez-Martín et al., 2012), which may impair the synthesis and repair of myelin and exacerbate the demyelination process (Poh et al., 2021). Research showed that quercetin can alleviate the extent of demyelination and the thickness decrease of myelin sheath, thereafter, enhancing the microglial engulfment ability of myelin fragments *in vitro* and *in vivo* (Tan et al., 2022). In this study, the cellular component (CC) terms identified by GO enrichment analysis of the hub genes enriched in extracellular space, caveola, cell surface, plasma membrane, extracellular matrix, neuron projection, and axon. The results combine the animal experiment, suggested that Epimedium can regulate the expression of cell adhesion molecules and MMPs by modulating the TNF signaling pathways. Our study also indicates that that Epimedium may exert its effects on VaD by regulating the BBB, alleviating demyelination and axonal damage which need further exploration.

## Conclusion

In summary, our study suggests that Epimedium exerts a protective effect on VaD. Epimedium can alleviate oxidative stress, neuroinflammation, BBB dysfunction, apoptosis, which may be achieved by regulating TNF signaling pathways. Although more detailed pharmacological mechanisms are needed to deeply explore the direct role of TNF signaling pathway in VaD. However, this study first revealed the mechanism of action of Epimedium in VaD systematically through a network pharmacological approach, which provides further theoretical basis for the use of Epimedium in the treatment of VaD.

## Data Availability Statement

The original contributions presented in the study are included in the article/Supplementary material, further inquiries can be directed to the corresponding author.

## Ethics Statement

The animal study was reviewed and approved by The Animal Experimentation Ethics Committee of the Second Affiliated Hospital of Chongqing Medical University.

## Author Contributions

CX designed and drafted the manuscript. CX and HT performed data analysis and data interpretation. CX and GL conducted the bioinformatics and statistical analyses. CL provided useful advice on the design of this study, and supervised the experimental work. All authors contributed to the article and approved the submitted version.

## Funding

This study was supported by the National Natural Science Foundation of China (Grant No. 81771248), the Medical Scientific Research Project of Chongqing Municipal Health commission (Grant No. 2018ZDXM022).

## Footnotes

^1^ http://tcmspw.com/

^2^ https://www.uniprot.org/

^3^ https://www.drugbank.ca

^4^ https://www.omim.org/

^5^ https://www.genecards.org/

^6^ http://db.idrblab.net/ttd/

^7^ https://www.disgenet.org/

^8^ https://bioinfogp.cnb.csic.es/tools/venny/index.html

^9^ https://string-db.org

^10^ https://david.ncifcrf.gov/

^11^ http://www.rcsb.org/

^12^ https://pubchem.ncbi.nlm.nih.gov/
